# Bioactive Phytochemicals from *Salix pseudolasiogyne* Twigs: Anti-Adipogenic Effect of 2′-*O*-Acetylsalicortin in 3T3-L1 Cells

**DOI:** 10.3390/ijms231912006

**Published:** 2022-10-09

**Authors:** Hee Jung Kim, Yoon Seo Jang, Ji Won Ha, Moon-Jin Ra, Sang-Mi Jung, Jeong-Nam Yu, Kyunga Kim, Ki Hyun Kim, Sung Hee Um

**Affiliations:** 1 Department of Molecular Cell Biology, Samsung Biomedical Research Institute, School of Medicine, Sungkyunkwan University, Suwon 16419, Korea; 2 Department of Health Sciences and Technology, Samsung Medical Center, Samsung Advanced Institute for Health Sciences and Technology, Sungkyunkwan University, Seoul 06351, Korea; 3 School of Pharmacy, Sungkyunkwan University, Suwon 16419, Korea; 4 Hongcheon Institute of Medicinal Herb, Hongcheon-gun 25142, Korea; 5 Nakdonggang National Institute of Biological Resources, Sangju 37242, Korea; 6 Biomedical Statistics Center, Research Institute for Future Medicine, Samsung Medical Center, Seoul 06351, Korea; 7 Biomedical Institute Convergence, Sungkyunkwan University, Suwon 16419, Korea

**Keywords:** *Salix pseudolasiogyne*, 2′-*O*-acetylsalicortin, adipogenesis, obesity, adipocyte differentiation

## Abstract

*Salix pseudolasiogyne* (Salicaceae) is a willow tree and has been used as a medicinal herb in Korea to treat pain and fever. As a part of an ongoing study to identify bioactive natural products, potential anti-adipogenic compounds were investigated using the ethanol (EtOH) extract of *S. pseudolasiogyne* twigs. Phytochemical investigation of the EtOH extracts using liquid chromatography–mass spectrometry (LC/MS) led to the separation of two compounds, oregonin (**1**) and 2′-*O*-acetylsalicortin (**2**). The structures of the isolates were identified using nuclear magnetic resonance spectroscopy and LC/MS analysis. To the best of our knowledge, it is the first report identifying oregonin (**1**) in twigs of *S. pseudolasiogyne*. Here, we found that the isolated compounds, oregonin (**1**) and 2′-*O*-acetylsalicortin (**2**), showed anti-adipogenic effects during 3T3-L1 cell differentiation. Notably, 2′-*O*-acetylsalicortin (**2**), at a concentration of 50 µM, significantly suppressed lipid accumulation. Moreover, the mRNA and protein levels of lipogenic and adipogenic transcription factors were reduced in 2′-*O*-acetylsalicortin (**2**)-treated 3T3-L1 cells. Taken together, these results indicate that 2′-*O*-acetylsalicortin (**2**), isolated from *S. pseudolasiogyne* twigs, has the potential to be applied as a therapeutic agent to effectively control adipocyte differentiation, a critical stage in the progression of obesity.

## 1. Introduction

Various bioactive natural compounds are derived from medicinal plants and they have been used as raw materials for the development of new drugs [1]. The genus *Salix* comprises approximately 400 species of deciduous trees and shrubs distributed across temperate regions of Asian countries, including Korea; willow trees are the most abundant among the *Salix* spp. [2]. For thousands of years, willow trees have been well known for effectively curing pain and inflammation since their bark was reported to possess anti-inflammatory metabolites, such as salicylic acid, which is a well-known natural source of aspirin [3,4]. In a similar context, previous pharmacological studies on *Salix* spp. have demonstrated diverse therapeutic effects, including anti-tumor [5], anti-inflammatory [6], anti-oxidant [7], and anti-obesity effects [8]. Specifically, salicin derivatives obtained from *Salix pseudolasiogyne* twigs were found to inhibit lipopolysaccharide-induced NO production in BV2 microglial cells in vitro, suggesting their potential anti-inflammatory effects [9]. *Salix pseudolasiogyne* H. Lev., also known as the weeping willow, belongs to the family *Salicaceae*, and has been used as a traditional Korean medicine for the treatment of pain, fever, and inflammation for thousands of years [10]. Although *S. pseudolasiogyne* is a promising resource for the discovery of natural bioactive products, bioactive phytochemicals from *S. pseudolasiogyne* have not been fully explored for controlling adipocyte differentiation.

Obesity is a pathological condition caused by excessive accumulation of adipose tissue in the body and is related to the prevalence of many health complications, such as type 2 diabetes, hypertension, heart failure, coronary heart disease, dyslipidemia, stroke, cancer, kidney disease, neurological disease, and cardiovascular diseases [11]. Adipose tissues play a crucial role in maintaining energy homeostasis all over the body, which involves an increase in the size and number of adipocytes and functional maturation through a process called adipogenesis [12]. The differentiation of preadipocytes, such as fibroblasts, into insulin-sensitive adipocytes accumulating mature lipids, is referred to as adipogenesis; it is considered to be a primary factor in obesity [13]. The great advancement of modern research on adipogenesis included the development of immortal preadipocyte cell lines, such as 3T3-L1 preadipocytes, which have been used extensively because they represent most of the main characteristics of adipocytes in vivo [14]. Birsoy et al. demonstrated that the differentiation of 3T3-L1 adipocytes showed a gene expression pattern similar to that of white adipose tissue (WAT) in vivo, providing direct evidence that the 3T3-L1 cell line exhibits many transcriptional programs that function during WAT development in vivo [15]. Adipocyte differentiation is regulated by various transcription factors, including peroxisome proliferator-activated receptor gamma (PPARγ) and CCAAT/enhancer binding protein (C/EBP) members δ, β, α, and many distinct signaling pathways such as hedgehog, Wnt, transforming growth factor-β, and adenosine monophosphate-activated protein kinase [16]. Hence, identifying a compound that inhibits adipocyte differentiation could potentially prevent or treat obesity.

As part of our continuing research to identify bioactive phytochemicals from diverse natural sources [17,18,19,20,21,22], we have examined the potential anti-adipogenic constituents present in an ethanol (EtOH) extract of *S. pseudolasiogyne* twigs. Phytochemical analyses of the EtOH extracts of *S. pseudolasiogyne* twigs combined with liquid chromatography–mass spectrometry (LC/MS)-based analysis led to the isolation of two compounds, oregonin (**1**) and 2′-*O*-acetylsalicortin (**2**). The structures of the isolated compounds (**1** and **2**) were determined using nuclear magnetic resonance (NMR) spectroscopy, physical data, and LC/MS analysis. Herein, we describe the isolation and structural elucidation of compounds (**1** and **2**) and evaluate their anti-adipogenic activities in 3T3-L1 cells and the potential mechanism of their action.

## 2. Results

### 2.1. Isolation and Identification of the Compounds

**Figure 1:**
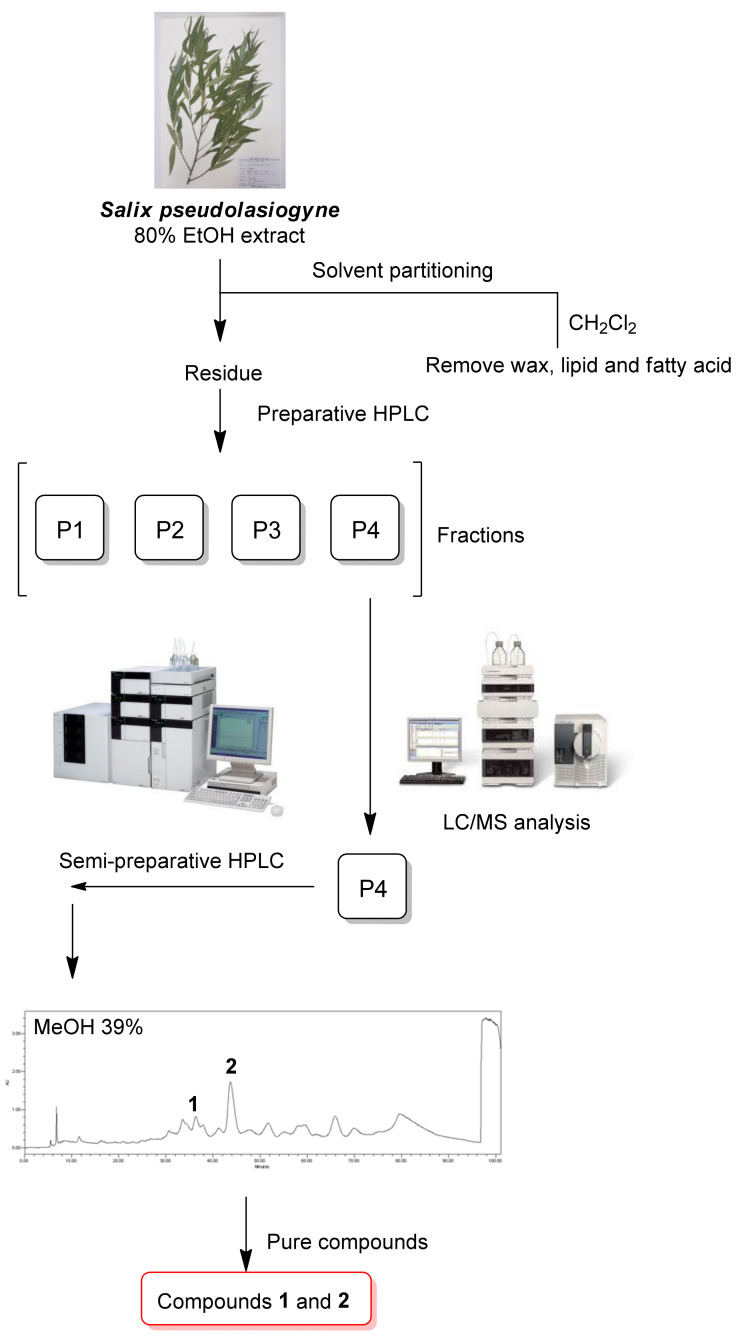
Schematic representation of oregonin (**1**) and 2′-*O*-acetylsalicortin (**2**) isolation.

**Figure 2:**
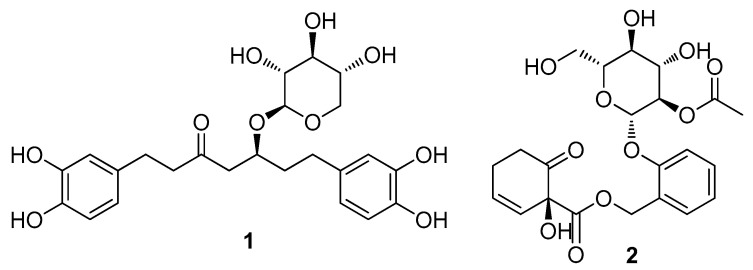
Chemical structure of oregonin (**1**) and 2′-*O*-acetylsalicortin (**2**).

Dried and pulverized *S. pseudolasiogyne* twigs were extracted three times using 80% aqueous EtOH and filtered at room temperature. The filtrate was concentrated to obtain a crude EtOH extract. Subsequently, to remove wax, lipids, and fatty acids, the extract was subjected to solvent partitioning with CH_2_Cl_2_; the resultant residue was fractionated by preparative reversed-phase high-performance liquid chromatography (HPLC) to obtain four fractions (P1–P4) (Figure 1 and Figure S3). The LC/MS analysis of the fractions obtained from preparative HPLC separation determined the presence of promising ion peaks with *m*/*z* 450–500 expected for the phenolic glycosides in fraction P4, which was subsequently purified by semi-preparative reversed-phase HPLC (Figure 1). HPLC separation allowed the isolation and identification of two compounds (**1** and **2**) (Figure 2). The chemical structures of compounds **1** and **2** were determined to be oregonin (**1**) and 2′-*O*-acetylsalicortin (**2**) by comparative analyses of NMR spectra (Figures S1 and S2) and physical data, including optical rotation values and the previously reported ones [23,24], and data from the LC/MS analysis of these compounds. To the best of our knowledge, this is the first study to identify oregonin (**1**) in *S. pseudolasiogyne*.

### 2.2. Effects of the Isolated Compounds on Intracellular Lipid Accumulation

**Figure 3:**
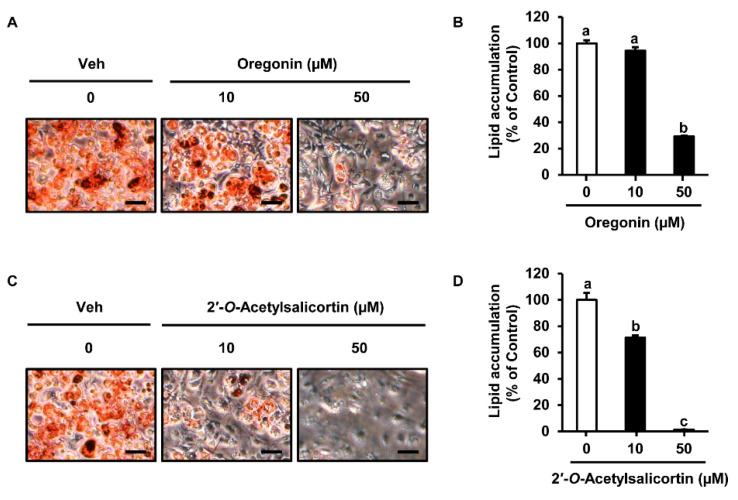
Inhibitory effect of oregonin and 2′-*O*-acetylsalicortin on intracellular lipid contents. The 3T3-L1 preadipocytes were induced to differentiate and then treated with oregonin. (**A**) After differentiation, Veh or oregonin (10 or 50 µM)-treated cells were stained with Oil Red. (**B**) Lipid contents were quantified by spectrophotometry (*n* = 3 per group). (**C**) Veh or 2′-*O*-acetylsalicortin (10 or 50 µM)-treated cells were stained with Oil Red O solution. (**D**) Intracellular lipid accumulation was analyzed by spectrophotometry (*n* = 3 per group). The values indicate the mean ± SEM. Mean values followed by different letters are significantly different (*p* ≤ 0.05). The size of each scale bar in photomicrographs is 100 µm. Veh, vehicle treatment (negative control).

According to a recent study, the MeOH extract of *S. pseudolasiogyne* twigs reduced lipid accumulation in 3T3-L1 preadipocytes, and the anti-adipogenic constituents were found to be salicortin derivatives [25]. We investigated the effects of the isolated compounds (**1** and **2**) on lipid accumulation in 3T3-L1 cells. The compounds were separately dissolved in dimethyl sulfoxide, and vehicle treatment (Veh) was used as a negative control. Cells were differentiated and treated with Veh, oregonin (10 and 50 µM), or 2′-*O*-acetylsalicortin (10 and 50 µM) for 7 days. Oil Red O-stained cells revealed significantly reduced lipid accumulation induced by 50 µM oregonin treatment compared to that in Veh-treated cells (Figure 3A). Oregonin (10 µM) treatment did not show a significant effect on lipid accumulation compared to that in Veh-treated cells. However, 50 µM oregonin lowered lipid accumulation by 71% compared with that in Veh-treated cells (Figure 3B). The treatments with 10 and 50 µM 2′-*O*-acetylsalicortin reduced the number of differentiated cells significantly compared with that in Veh-treated cells (Figure 3C). Additionally, lipid accumulation was inhibited by 10 µM 2′-*O*-acetylsalicortin and up to 98% in 50 µM 2′-*O*-acetylsalicortin-treated cells compared to that in Veh-treated cells (Figure 3D). Both compounds (**1** and **2**) showed an inhibitory effect on lipid accumulation when used at a concentration of 50 µM; however, only 2′-*O*-acetylsalicortin effectively reduced lipid accumulation even at a low concentration (10 µM).

### 2.3. 2′-O-Acetylsalicortin Attenuates Adipogenesis by Inhibiting the Expression of Adipogenic Transcription Factors and Lipogenic Factors

**Figure 4:**
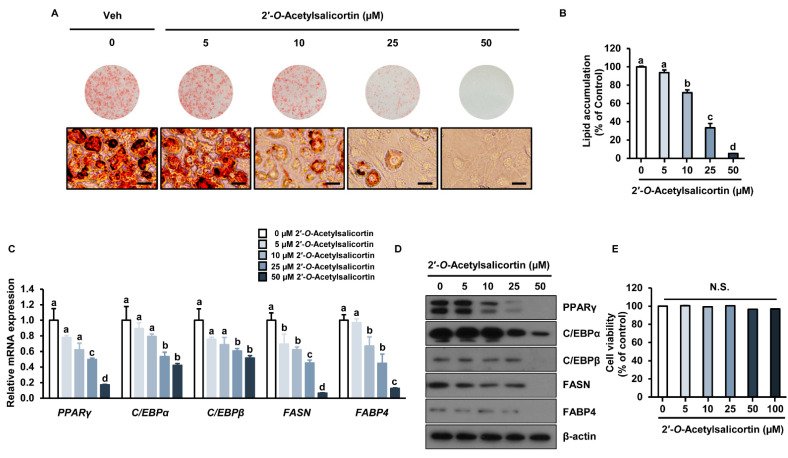
Inhibitory effect of 2′-*O*-acetylsalicortin on adipogenesis. (**A**) 3T3-L1 cells treated with various concentrations of 2′-*O*-acetylsalicortin during the adipocyte differentiation were stained with Oil Red O (upper) and analyzed by microscopy (bottom). (**B**) Quantification of intracellular lipid contents with spectrophotometry (*n* = 3 per group). (**C**) Expressions of *PPARγ*, *FABP4*, *FASN*, *C/EBPα*, and *C/EBPβ* genes were examined using qRT-PCR (*n* = 3 per group). (**D**) Protein levels of C/EBPβ, FASN, FABP4, C/EBPα, and PPARγ were analyzed by immunoblotting. (**E**) Cytotoxic effect of 2′-*O*-acetylsalicortin (*n* = 3 per group). The values represent the mean ± SEM. The size of each scale bar in photomicrographs is 100 µm. Mean values followed by different letters are significantly different (*p* ≤ 0.05). N.S., not significant. Veh, vehicle treatment (negative control).

We further conducted detailed analyses of the inhibitory effect of various concentrations of 2′-*O*-acetylsalicortin. Oil Red O staining revealed that lipid content was reduced in 10, 25, and 50 µM 2′-*O*-acetylsalicortin-treated cells compared with that in vehicle-treated cells (Figure 4A). Lipid accumulation was also inhibited by 29%, 67%, and 94% upon treatment with 10, 25, and 50 µM 2′-*O*-acetylsalicortin, respectively, compared to that in Veh-treated cells (Figure 4B). Furthermore, mRNA expressions of adipogenic transcription factor *PPARγ* as well as lipogenic factors such as fatty acid synthase (*FASN*) and fatty acid binding protein 4 (*FABP4*) were significantly reduced by 10, 25, and 50 µM of 2′-*O*-acetylsalicortin (Figure 4C). The mRNA levels of *C/EBPα* and *C/EBPβ* were decreased in cells treated with 25 or 50 µM 2′-*O*-acetylsalicortin (Figure 4C). The protein levels of C/EBPβ, FASN, FABP4, C/EBPα, and PPARγ were also reduced in cells treated with 25 or 50 µM 2′-*O*-acetylsalicortin (Figure 4D). To determine if cytotoxicity is associated with the inhibitory effect of 2′-*O*-acetylsalicortin on adipogenesis, we performed an MTT assay in 3T3-L1 cells. However, the cell viability was not affected by 2′-*O*-acetylsalicortin at concentrations of up to 100 µM (Figure 4E). These results suggest that 2′-*O*-acetylsalicortin reduces lipid accumulation by inhibiting the mRNA and protein levels of lipogenic enzymes and adipogenic transcription factors, resulting in suppressed adipocyte differentiation.

## 3. Discussion

*S. pseudolasiogyne*, the willow tree used in this study, is common in several Asian countries and used in Korean traditional medicine to treat fever and pain [10]. Although salicin derivatives obtained from *S. pseudolasiogyne* exert anti-inflammatory effects [9], the effect of 2′-*O*-acetylsalicortin isolated from *S. pseudolasiogyne* twigs on adipogenesis has not been fully elucidated. 2′-*O*-acetylsalicortin, a bioactive phytochemical identified in *S. pseudolasiogyne* twigs, is a salicin derivative with a 1-hydroxy-6-oxo-2-cyclohexenecarboxylate moiety and an acetyl group in its sugar unit at C-2′. To date, other analogs of salicortin with acetyl groups in the sugar unit have been chemically identified, which include 6′-*O*-acetylsalicortin, 2′,6′-*O*-acetylsalicortin, and 3′-*O*-acetylsalicortin [9]. Here, we have revealed the inhibitory effect of 2′-*O*-acetylsalicortin on adipocyte differentiation.

Our results showed that 2′-*O*-acetylsalicortin at up to 50 µM concentrations reduced the triglyceride content of adipocytes without cytotoxic effects. Moreover, we detected that 2′-*O*-acetylsalicortin treatment attenuated the mRNA levels of adipogenic and lipogenic factors in 3T3-L1 cells. The protein levels of PPARγ, FASN, FABP4, C/EBPβ, and C/EBPα also decreased upon treatment with 2′-*O*-acetylsalicortin. Adipocyte differentiation is accompanied by the accumulation of lipids and an increased number of adipocytes, which is closely related to the initiation of obesity [25]. The expression of PPARγ and C/EBPs families, the major transcription factors stimulating adipogenesis, as well as lipogenic factors, including FASN and FABP4, play important roles in adipocyte differentiation [26]. Considering that the 2′-*O*-acetylsalicortin suppressed mRNA and protein levels of major adipogenic transcription factors and lipogenic factors, we infer that this compound is a potential agent for the development of anti-obesity drugs.

Similarly, polyphenol fractions extracted from *Salix matsudana* leaves significantly prevented high-fat diet-induced obesity in mice and reduced hepatic total cholesterol content; however, the exact bioactive compound that affected anti-obesity was not identified [8]. Furthermore, the EtOH extract derived from *Salix babylonica* L. leaves significantly inhibited the elevation of triacylglycerol content in the blood along with weight loss and led to a decrease in adipose tissue weight in high-fat diet-fed mice [27]. Although EtOH extracts from Salicis Radicis Cortex (root bark of *Ulmus davidiana* var. *japonica*) inhibited lipid accumulation in 3T3-L1 cells, the molecular targets and precise components responsible for this effect have not been investigated [28]. Additionally, previous phytochemical studies on *S. pseudolasiogyne* detected the presence of the primary constituents of salicin and some salicin derivatives, including 2′-*O*-acetylsalicin, salicortin, 2′-*O*-acetylsalicortin, 3′-*O*-acetylsalicortin, 6′-*O*-acetylsalicortin, and 2′,6′-*O*-acetylsalicortin [29]. However, only 2′-*O*-acetylsalicortin was found to have an IC_50_ value of 24.6 µM for the inhibition of adipocyte differentiation, and the detailed molecular mechanisms were not studied [29]. Our results revealed that 10–50 µM 2′-*O*-acetylsalicortin isolated from *S. pseudolasiogyne* twigs reduced intracellular lipid accumulation in a dose-dependent manner and lowered the mRNA and protein levels of lipogenic enzymes and adipogenic factors. These results suggest that 2′-*O*-acetylsalicortin isolated from *S. pseudolasiogyne* twigs can be developed as a potential anti-obesity agent. As salicortin derivatives exhibit an anti-inflammatory effect through inhibition of NF-κB and JNK MAPK signaling, future studies are required to investigate whether 2′-*O*-acetylsalicortin, a salicortin derivative, affects adipocyte differentiation through these signaling pathways.

**Figure 5:**
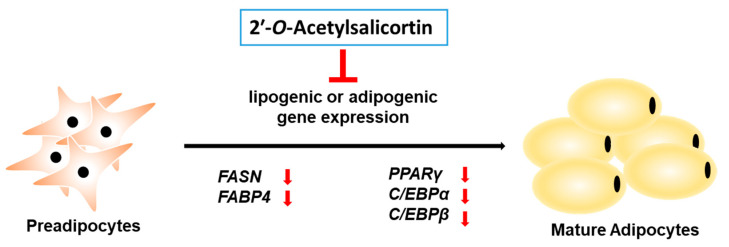
The model on the action of the 2′-*O*-acetylsalicortin in 3T3-L1 differentiation. 2′-*O*-acetylsalicortin represses adipocyte differentiation through the downregulation of mRNA and proteins of lipogenic enzymes and adipogenic factors.

For a long period, plants have been traditionally utilized to prevent or treat obesity, and several bioactive compounds from edible plants have been isolated for the development and discovery of novel anti-obesity drugs [30]. Plant-derived molecules, such as cellatrol and wedaferrin A, improve leptin sensitivity, leading to weight loss [31,32]. Many medicinal plants and their bioactive compounds have been clinically tested and recognized as effective drug candidates for treating obesity and type 2 diabetes [33]. For example, *Rhinacanthus nasutus* (L.) Kurz (Acanthaceae), a common medicinal herb found in Thailand and Southeast Asia, has been traditionally used to treat type 2 diabetes because it has displayed anti-adipogenic, anti-hyperglycemic, and anti-hyperlipidemic effects [34,35,36]. In addition, among all such plants, the genus *Salix* has been identified as a crucial medicinal herb because it shows important anti-inflammatory activities due to the presence of salicin, which is a precursor of acetylsalicylic acid [9,37]. They also show anti-cancer, antidiabetic, antioxidant, anti-inflammatory, antimicrobial, hepatoprotective, and neuroprotective activities [38]. In addition, the MeOH extract of *S. pseudolasiogyne* was determined to display a cognitive-enhancing effect on scopolamine-induced memory deficit in mice, and it reduced glutathione reductase activity [9]. Moreover, 2′-*O*-acetylsalicortin from this MeOH extract showed an anti-amnesic activity through a significant reversal of the reduced antioxidant activities of enzymes, such as superoxide dismutase, glutathione peroxidase, glutathione reductase, and glutathione content [9]. The current study elucidated that 2′-*O*-acetylsalicortin suppressed the development of preadipocytes into mature adipocytes by reducing the levels of mRNAs and proteins affecting lipogenesis and adipogenesis (Figure 5). Therefore, our results suggest that 2′-*O*-acetylsalicortin, a natural bioactive product, can be a potent drug candidate for the prevention and treatment of obesity.

## 4. Materials and Methods

### 4.1. General Experimental Procedures

Optical rotation was measured using a Jasco P-1020 polarimeter (Jasco, Easton, MD, USA). Ultraviolet (UV) spectra were acquired on an Agilent 8453 UV-visible spectrophotometer (Agilent Technologies, Santa Clara, CA, USA). IR spectra were recorded using a Bruker IFS-66/S FT-IR spectrometer (Bruker, Karlsruhe, Germany). Electrospray ionization (ESI) mass spectra were recorded on an Agilent 1200 Series HPLC system (Agilent Technologies, Santa Clara, CA, USA), equipped with a diode array detector and 6130 Series ESI mass spectrometer using an analytical Kinetex C_18_ 100 Å column (100 × 2.1 mm, 5 μm; Phenomenex, Torrance, CA, USA; solvent condition: from 10% MeOH/H_2_O to 100% MeOH (gradient system, 0–20 min); flow rate: 0.3 mL/min). NMR spectra were recorded using a Bruker AVANCE III 850 NMR spectrometer operating at 850 MHz (^1^H) and 212.5 MHz (^13^C) (Bruker, Karlsruhe, Germany), with chemical shifts indicated in ppm (δ). For preparative HPLC, we used a Waters 1525 Binary HPLC pump with Waters 996 Photodiode Array Detector (Waters Corporation, Milford, CT, USA). Semi-preparative HPLC was performed using a Shimadzu Prominence HPLC System with SPD-20A/20AV Series Prominence HPLC UV-Vis Detectors (Shimadzu, Tokyo, Japan). Merck precoated silica gel F254 and RP-18 F254s plates (Merck, Darmstadt, Germany) were used for TLC; spots were detected using TLC under UV light or by spraying anisaldehyde–sulfuric acid followed by heating.

### 4.2. Plant Material

*S. pseudolasiogyne* twigs were collected in Chungcheongnam-do, Korea, in June 2021. A voucher specimen (HIMH-2109) was identified by Dr. Hye-Ryen Na at the Northeastern Asia Biodiversity Institute, Seoul 05677, Korea. The material was kept in the herbarium of Nakdonggang National Institute of Biological Resources, Sangju, Korea.

### 4.3. Extraction and Isolation

*S. pseudolasiogyne* twigs (1.6 kg) were dried at 35–45 °C in an oven for one week, pulverized, and sonicated with 80% EtOH (10 L) for 90 min three times at room temperature. The resulting EtOH extract was evaporated in vacuo to obtain a crude brown EtOH extract (123.6 g). The extract (9.1 g) was dissolved in distilled water (700 mL) for solvent partitioning using CH_2_Cl_2_ to remove wax, lipids, and fatty acids, and the residue was concentrated using an evaporator to yield the crude extract (4.0 g). The crude extract (1.0 g) was separated using preparative reversed-phase HPLC (from 30% MeOH to 50% MeOH for 80 min, gradient system) to obtain four fractions (P1–P4). Fraction P4 (306 mg) was isolated by semi-preparative reversed-phase HPLC using 39% MeOH to obtain compounds **1** (0.7 mg, *t*_R_ = 43.0 min, ESIMS (positive-ion mode) *m*/*z* 479 [M + H]^+^) and **2** (5.3 mg, *t*_R_ = 47.5 min, ESIMS (positive-ion mode) *m*/*z* 467 [M + H]^+^).

### 4.4. 3T3-L1 Cell Culture and Adipocyte Differentiation

Dulbecco’s modified Eagle’s medium (DMEM) with streptomycin–penicillin (1%) and bovine serum (10%) were used to culture 3T3-L1 preadipocytes. The 3T3-L1 cell line was obtained from the American Type Culture Collection (ATCC). For adipocyte differentiation, the culture medium was changed to DMEM containing 1 µM dexamethasone, 10% fetal bovine serum (FBS), 5.0 µg/mL insulin, and 0.5 mM IBMX. Thereafter, it was replaced with DMEM, FBS (10%), and insulin after two days of differentiation. The medium was changed every two days until seven days of adipocyte differentiation [39].

### 4.5. Oil Red O Staining

The cells were stained with Oil Red O solution 7 days after the differentiation started. The cells were washed three times with phosphate-buffered saline (PBS) and fixed using formalin (10%). The cells were then incubated with 10% formalin for 1 h. The cells were incubated with isopropanol (60%) for 5 min and dried completely. The cells were treated with Oil Red O (Sigma, St. Louis, MO, USA, cat. O0625) for at least 10 min and washed thoroughly with distilled water (five times). For the quantification of lipid droplets, 100% isopropanol was used to dissolve the stained cells, which were then used in spectrophotometric analysis (wavelength 500 nm) [40].

### 4.6. Extraction of Protein and Western Blotting

Proteins were extracted from 3T3-L1 cells. For this purpose, cells were rinsed with PBS (ice-cold); dissolved in a radioimmunoprecipitation assay buffer supplemented with 2 mM Na_3_VO_4_, 0.1 M NaF, 0.25% deoxycholate, and protease inhibitor cocktail (Roche, Basel, Switzerland, cat. 11697498001) for 30 min; and then centrifuged. The Bio-Rad protein assay dye reagent (Bio-Rad, Hercules, CA, USA, cat. 500-0006) was used to calculate the protein concentration using the Bradford method. Protein molecules were separated using sodium dodecyl sulfate–polyacrylamide gel electrophoresis followed by transfer to polyvinylidene difluoride membranes and blocking with 5% bovine serum albumin for 1 h. Incubation with primary antibodies against C/EBPα, C/EBPβ, FASN, FABP4, and PPARγ was done overnight at 4 °C. The membrane was then incubated with secondary antibody. Tris-buffered saline with Tween 20 (0.1%) detergent was used to wash the membranes 3 times. The ECL system (Thermo Fisher Scientific, Waltham, MA, USA, cat. 34580) was used to detect protein signals.

### 4.7. RNA Isolation and Quantitative Polymerase Chain Reaction (qPCR)

Total RNA was isolated using TRIzol reagent (Thermo Fisher Scientific, cat. 15596026). For complementary DNA (cDNA) synthesis, 1 µg of RNA was mixed with random hexamer primers and SuperScript II reverse transcriptase, followed by PCR, according to instructions provide by the manufacturer. Quantitative polymerase chain reaction (qPCR) was performed using cDNA as a template. The primers that were included in this analysis are listed in Table 1.

### 4.8. Statistical Analysis

Data are presented as mean ± standard error of the mean (SEM). Student’s *t*-test (un-paired, two-tailed) was used to analyze the main and interaction effects. A two-tailed *t*-test was conducted to estimate the differences between individual group means. Data with different letters are considered statistically different (*p* < 0.05).

## 5. Conclusions

In conclusion, phytochemical investigation of the EtOH extract of *S. pseudolasiogyne* twigs led to the isolation and identification of two compounds, oregonin (**1**) and 2′-*O*-acetylsalicortin (**2**) in the process of discovery of potential anti-adipogenic compounds. To the best of our knowledge, this is the first study to identify oregonin (**1**) in *S. pseudolasiogyne*. The pharmacological study demonstrated that 2′-*O*-acetylsalicortin (**2**) reduced lipid accumulation in 3T3-L1 cells. Moreover, it decreased the mRNA and protein levels of adipogenic and lipogenic transcription factors during adipogenesis. Hence, 2′-*O*-acetylsalicortin (**2**) could be a potential therapeutic agent to inhibit the progression of adipocyte differentiation leading to obesity.

## Figures and Tables

**Table 1:** List of primers used for real-time qPCR.

Name	Forward (5′–3′)	Reverse (5′–3′)
*C/EBPα*	ACAACATCGCGGTGCGCAAGA	TGCCATGGCCTTGACCAAGGAG
*C/EBPβ*	GTCCAAACCAACCGCACAT	CAGAGGGAGAAGCAGAGAGTT
*PPARγ*	GGGTGAAACTCTGGGAGATTCTCC	CAGCAACCATTGGGTCAGCTCT
*FABP4*	TGG AAG CTT GTC TCC AGT GA	AAT CCC CAT TTA CGC TGA TG
*FASN*	CGGAAACTGCAGGAGCTGTC	CACGGAGTTGAGCCGCAT
*GAPDH*	GTCTTCCTGGGCAAGCAGTA	CTGGACAGAAACCCCACTTC

## Data Availability

Not applicable.

## Supplementary Materials

The following supporting information can be downloaded at: https://www.mdpi.com/article/10.3390/ijms231912006/s1.
