# Supplementary material for: Bioactive Phytochemicals from Salix pseudolasiogyne Twigs: Anti-Adipogenic Effect of 2′-O-Acetylsalicortin in 3T3-L1 Cells
Source: Int J Mol Sci. 2022 Oct 9;23(19):12006. doi: 10.3390/ijms231912006 (PMC9570486; doi:10.3390/ijms231912006)

Supplementary data

Bioactive Phytochemicals from *Salix pseudolasiogyne* Twigs: Anti-Adipogenic Effect of 2′-*O*-Acetylsalicortin in 3T3-L1 Cells

Hee Jung Kim [1,2,†], Yoon Seo Jang [3,†], Ji Won Ha [3], Moon-Jin Ra [4], Sang-Mi Jung [4], Jeong-Nam Yu [5], Kyunga Kim [6], Ki Hyun Kim [3,*] and Sung Hee Um [1,2,7,*]

[1] Department of Molecular Cell Biology, Samsung Biomedical Research Institute, School of Medicine, Sungkyunkwan University, Suwon 16419, Korea
[2] Department of Health Sciences and Technology, Samsung Medical Center, Samsung Advanced Institute for Health Sciences and Technology, Sungkyunkwan University, Seoul 06351, Korea
[3] School of Pharmacy, Sungkyunkwan University, Suwon 16419, Korea
[4] Hongcheon Institute of Medicinal Herb, Hongcheon-gun 25142, Korea
[5] Nakdonggang National Institute of Biological Resources, Sangju 37242, Korea
[6] Biomedical Statistics Center, Research Institute for Future Medicine, Samsung Medical Center, Seoul 06351, Korea
[7] Biomedical Institute Convergence, Sungkyunkwan University, Suwon 16419, Korea
* Correspondence: khkim83@skku.edu (K.H.K.); shum@skku.edu (S.H.U.); Tel.: +82-31-290-7700 (K.H.K.); Tel.: +82-31-299-6123 (S.H.U.)
† These authors contributed equally to this work.

Figure S1. ^{1}H NMR spectrum of oregonin (**1**) (CD_3OD, 850 MHz)

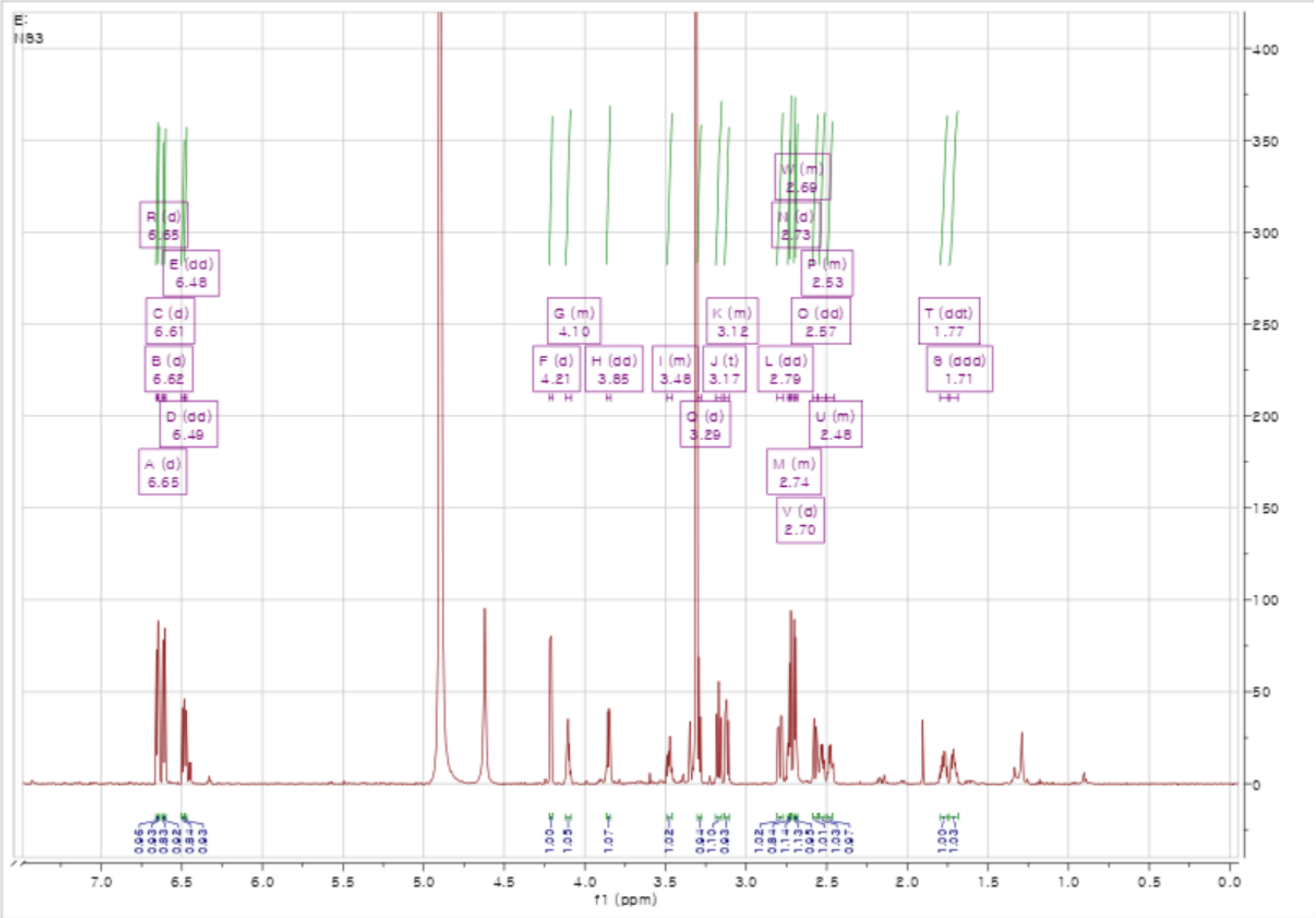

Figure S2. ^{1}H NMR spectrum of 2′-*O*-acetylsalicortin (**2**) (CD_3OD, 850 MHz)

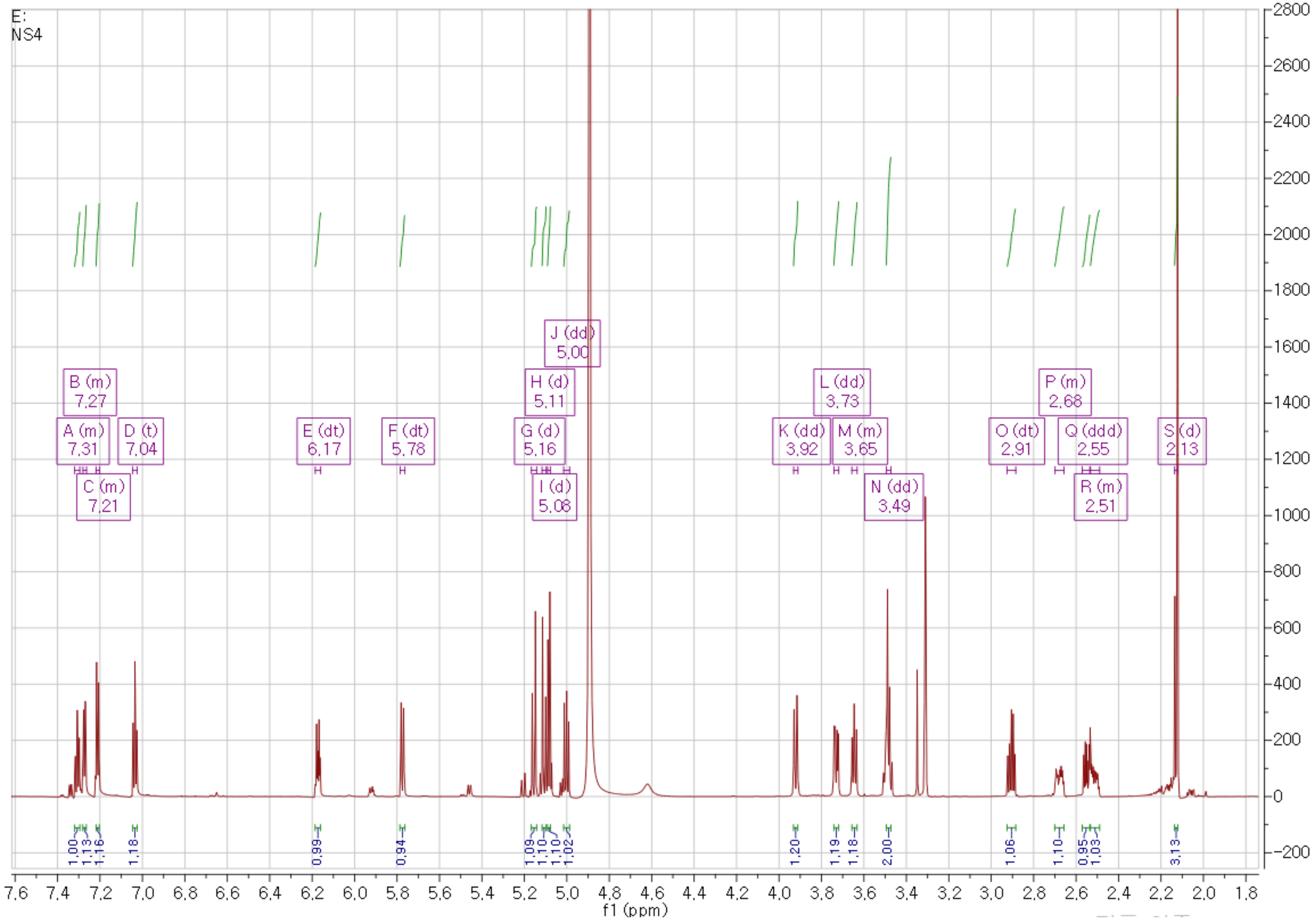

Figure S3. HPLC chromatogram of fractions (P1 – P4).

- Column : C18
- Solvent : MeOH Gradient 30~50%
- Sample Wt : 1.0 g

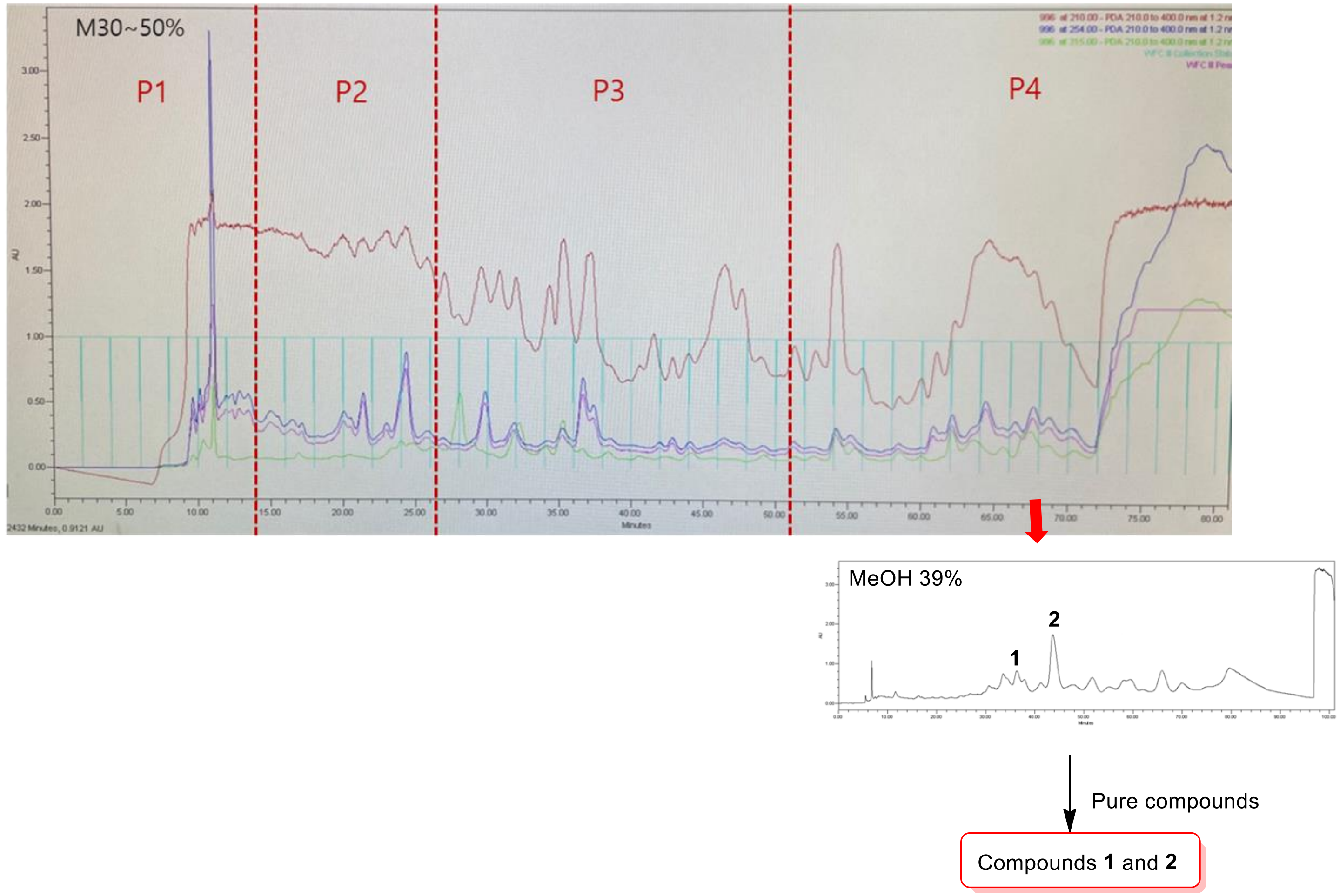

Supplement: Supplementary file 1 [file ijms-23-12006-s001.zip › ijms-1938227-supplementary.pdf]
